# Disparities in Access to Revascularization: Evidence from New York

**DOI:** 10.1089/heq.2018.0073

**Published:** 2019-08-28

**Authors:** Michael K. Gusmano, Daniel Weisz, Catherine Allende, Victor G. Rodwin

**Affiliations:** ^1^ Department of Health Behavior, Society and Policy, Rutgers University School of Public Health, Piscataway, New Jersey.; ^2^ Department of Research, The Hastings Center, Garrison, New York.; ^3^ Robert N. Butler Center for Aging, Columbia University, New York, New York.; ^4^ Memorial Sloan Kettering Cancer Center, New York, New York.; ^5^ Wagner School of Public Service, New York University, New York, New York.

**Keywords:** revascularization, New York City, gender disparities

## Abstract

**Purpose:** To quantify and compare citywide disparities in the performance of coronary revascularization procedures in New York residents diagnosed with ischemic heart disease (IHD) by the characteristics of the patients and their neighborhood of residence in 2000–2002 and 2011–2013.

**Methods:** We identify the number of hospitalizations for patients with diagnoses of IHD and/or congestive heart failure (CHF) and the number of revascularization procedures performed on the population 45 years and older, relying on hospital administrative data for New York City, by area of residence, from the Statewide Planning and Research Cooperative System (SPARCS). We conduct multiple logistic regressions to analyze the factors associated with revascularization for hospitalized patients admitted with IHD and CHF over the two time periods.

**Results:** Despite any decline in population health status, both the age-adjusted rates of inpatient hospital discharges for acute myocardial infarction, for IHD and for CHF, decreased as did the rates of revascularization procedures. Racial and ethnic disparities were much smaller in the later period than those documented earlier. However, there were persistent gender, insurance status, and neighborhood-level disparities in the treatment of heart disease.

**Conclusions:** With the declines in rates of heart disease, our findings point to the need for more clinical and population-based research to improve the understanding of why race/ethnicity, gender, insurance status, and neighborhood-level disparities persist in the treatment of heart disease.

## Introduction

There has been a striking decline in coronary artery disease (CAD) mortality over the past four decades, but it remains the leading cause of death in the United States.^1,2^ Much of this decline is attributable to public health interventions, particularly reductions in tobacco use^3^ as well as education strategies related to hypertension, diabetes, avoiding dietary trans fat, and obesity. Along with these efforts, the treatment of heart disease has also contributed to reductions in mortality.^4–6^ For many patients with CAD, the provision of anti-antigal medications and medications to modify atherosclerosis has improved outcomes.^7^ For other patients with obstructive CAD, revascularization, in either the form of a coronary artery bypass graft (CABG) or a percutaneous transluminal coronary intervention (PTCI), often using drug eluting stents is an important treatment option.^8^

Revascularization is widely recognized as an effective treatment for patients with unstable CAD, as well as for those with stable ischemic heart disease (IHD) with progressive or refractory symptoms. Despite increasing reliance on guideline-directed medical therapy, previous research has found that women, racial and ethnic minorities, patients without health insurance, and those who live in low-income neighborhoods may have inadequate access to these procedures.^9^ An analysis of access to revascularization in Manhattan between 1997 and 2000 found large and significant disparities associated with these factors in the use of revascularization procedures among patients hospitalized with obstructive CAD (captured by including patients with diagnoses of IHD and/or congestive heart failure [CHF]).^10^

Here, based on analysis for the period 2011–2013, we examine whether disparities in access to revascularization in New York City (NYC) have narrowed. We compare how the use of revascularization procedures has evolved among patients, 45 years and older, hospitalized with IHD or CHF since 2000–2002, the period corresponding with Mayor Michael Bloomberg's tenure in office (2001–2013).

The Bloomberg administration adopted several policies and programs to reduce disparities in population health and in access to health care in NYC. Many of their prominent efforts aimed at reducing risk factors for heart disease. These included the ban on tobacco use in all commercial establishments, the ban on trans fats in all restaurants, the mandatory posting of calorie counts in chain restaurants, and the failed attempt to ban the sale of sugared beverages more than 16 ounces.^11,12^ The city's Department of Health and Mental Hygiene (DOHMH) established the New York City Community Health Survey (NYCCHS) in 2002, the nation's first community-based health and nutrition survey and a host of other efforts to track chronic illness.^12^ DOHMH uses data on health behaviors and outcomes to target programs and reduce population health and health care disparities. In 2004, DOHMH launched Take Care New York (TCNY) to reduce mortality from causes amenable to public health and health care interventions. TCNY started with 10 priority areas and 16 indicators.^13^ It has continued beyond the Bloomberg administration and now includes a range of interventions designed to improve population health and reduce health care disparities. The city's smoking ban is noteworthy because heart disease mortality, as well as hospital admissions for acute myocardial infarction (AMI), IHD, and CHF decreased between 2002 and 2013, despite an increase in several well-known risk factors, including obesity, hypertension and high cholesterol (Tables 1 and 2).

**Table 1.** New York City Population Characteristics

	**2002**	**2013**
Population		
Total population 45–64	1,695,148^a^	1,997,388^b^
Total population 65+	939,370^a^	993,158^b^
Total heart disease mortality rate per 100,000	321.59^a^	208.63^b^
Risk factors for heart disease, %		
Have you ever been told that you have high cholesterol?	26.2^c^	30.0^d^
Have you ever been told that you have high blood pressure?	25.9^c^	29.1^d^
Self-reported general health status fair or poor	19.5^c^	23.1^d^
Overweight	35^c^	32.7^d^
Obese	18.2^c^	23.4^d^
Overweight or obese	53.2^c^	56.1^d^

Age adjustment based on the 2000 U.S. Census population.

^a^ 2000 U.S. Census.

^b^ 2010 U.S. Census.

^c^ 2002 New York City Community Health Survey.

^d^ 2013 New York City Community Health Survey.

**Table 2.** Age-Adjusted Rates of Heart Disease Hospitalization and Treatment in New York City, 2000–2002 and 2011–2013

	2000–2002		2011–2013	
	45–64	65+	45–64	65+
Age-adjusted^a^ rate of hospital inpatient discharges for acute myocardial infarction per 100,000	274.8	1017.3	190.9	628.5
Age-adjusted^a^ rate of hospital inpatient discharges for all ischemic heart diseases per 100,000	1046.6	2880.6	649.4	1804.7
Age-adjusted^a^ rate of hospital inpatient discharges for all congestive heart failures per 100,000	394.4	2270.6	288.7	1942.1
Age-adjusted^a^ rate of revascularization per 100,000	474.8	1173.9	364.0	971.3
Age-adjusted^a^ rate of coronary artery bypass graft surgery per 100,000	132.6	396.5	58.5	172.1
Age-adjusted^a^ rate of percutaneous transluminal coronary angioplasty per 100,000	343.7	782.2	307.0	803.3

Source: New York State, SPARCS, 2000–2002 and 2011–2013.

^a^ Age adjustment based on the 2000 U.S. Census population.

SPARCS, Statewide Planning and Research Cooperative System.

An analysis of access to primary care in NYC found that gender, insurance status, racial, ethnic, and neighborhood-level disparities did not change over the course of the Bloomberg administration.^14^ Our findings on revascularization are similar. Although there were notable reductions in racial and ethnic disparities in the use of revascularization procedures, gender, insurance status, and neighborhood-level differences were conspicuously stable over the time periods we examined.

## Methods

### Data

To identify the number of hospitalizations for patients with diagnoses of IHD and/or CHF and the number of revascularizations performed on the population 45 years and older, we rely on hospital administrative data for NYC, by area of residence, from the Statewide Planning and Research Cooperative System (SPARCS), a comprehensive inpatient hospital patient data system established in 1979 by the New York State Department of Health in cooperation with hospitals. To assure an adequate number of hospital discharges and procedures for statistically meaningful comparisons, we calculate two 3-year averages for 2000–2002 and 2011–2013. To calculate age-specific and age-adjusted population rates, we rely on U.S. Census data for 2000 and 2010 and weights derived from the 2000 U.S. Census.

### Statistical analysis

We conducted multiple logistic regressions to analyze the factors associated with revascularization for hospitalized patients admitted with IHD and CHF over two time periods: 2000–2002 and 2011–2013. Both models estimate the probability that patients, ages 45 years and older with these discharge diagnoses, would receive a revascularization procedure (PTCI or CABG). The independent individual variables are age, gender, race/ethnicity, primary payers, and number of diagnoses on record (as a measure of illness severity). The neighborhood-level variables (by zip code) to assess access to these services include median household income quartile, and physician density (Andersen 1995). Drawing on Andersen's terminology, age, gender, race/ethnicity, and income are “predisposing” characteristics; insurance status, physician density, and zip code of residence are “enabling” characteristics.^15^

We include the variable, “age squared,” in our models, in addition to continuous age variables, because the probability of revascularization increases between the ages of 45 and 75 and decreases thereafter due to increasing frailty. Because observations on individuals from the same neighborhood may be correlated, we tested for bias due to unobserved neighborhood-level heterogeneity, by estimating the models with a dummy variable for each zip code as a replacement for neighborhood-level variables. The parameter estimates for the individual characteristics were not appreciably different from those generated by the original model.

## Results

Over the 2002 and 2013 period, data from the NYCCHS indicate that risk factors associated with heart disease increased (Table 1). There was a rise in prevalence of the population with high cholesterol and high blood pressure. Similarly, there was an increase in the obese population as well as the share of population reporting fair or poor health. Despite the decline in population health status, the age-adjusted rate of inpatient hospital discharges for both AMI and for IHD/CHF decreased (Table 2). These changes are comparable with those at the national level.^16,17^

Overall, for the population cohort aged 45–64 years, the age-adjusted rate of revascularization among patients hospitalized with IHD or CHF decreased by about 23%. Among patients 65 years and older, the rate of revascularization decreased by about 17% (Table 2). Most of this decline was in the age-adjusted rate of CABG. Among patients 45–64 years of age, the rate of CABG fell by nearly 56% and among patients 65 years and older it fell by nearly 57%. In contrast, the age-adjusted rate of PTCI fell by about 10.6% among patients, ages 45–64 years, and *increased* by about 2.7% among patients 65 years and older.

We found that, in both time periods, insurance status, race, gender, number of diagnoses, and zip code of residence are all associated with statistically significant odds ratios for revascularization (Tables 3 and 4). Residents in the low-income zip-code quartiles are less likely to receive revascularization than those in high-income zip-code quartiles. The odds of women, hospitalized with IHD or CHF, receiving revascularization were about 37% lower than among men during the 2000–2002 period (Table 3) and 35% lower than among men during the 2011–2013 period (Table 4).

**Table 3.** Logistic Regression Predicting Revascularization Among Patients Age 45+ with Ischemic Heart Disease or Congestive Heart Failure, New York City, 2000–2002

	*B*	SE	Wald	DF	Sig	Exp. (*B*)	95% Confidence interval	
							Lower	Upper
Age	0.241	0.005	2522.842	1	0.000	1.273	1.261	1.285
Age squared	−0.002	0.000	3594.429	1	0.000	0.998	0.998	0.998
Female (male)	−0.470	0.010	2265.382	1	0.000	0.625	0.613	0.637
Non-Hispanic black (NH white)	−1.391	0.017	6545.371	1	0.000	0.249	0.240	0.257
Hispanic (NH white)	−0.802	0.018	2029.171	1	0.000	0.448	0.433	0.464
Asian/other (NH white)	−0.215	0.014	232.259	1	0.000	0.806	0.784	0.829
Lowest income neighborhoods (highest income neighborhoods)	−0.267	0.015	300.186	1	0.000	0.766	0.743	0.789
Second quartile income neighborhoods (highest income neighborhoods)	−0.218	0.013	270.226	1	0.000	0.804	0.784	0.826
Third quartile income neighborhoods (highest income neighborhoods)	−0.110	0.013	75.224	1	0.000	0.896	0.874	0.918
Uninsured (private insurance)	−0.897	0.032	801.978	1	0.000	0.408	0.383	0.434
Medicare (private insurance)	−0.354	0.013	766.914	1	0.000	0.702	0.684	0.720
Medicaid (private insurance)	−0.458	0.015	941.164	1	0.000	0.633	0.614	0.651
Other insurance (private insurance)	−0.454	0.068	44.268	1	0.000	0.635	0.556	0.726
Number of diagnoses on record	−0.129	0.002	5482.099	1	0.000	0.879	0.876	0.882
Total physicians 1000 population	0.007	0.001	42.465	1	0.000	1.007	1.005	1.009
Constant	−6.639	0.161	1708.227	1	0.000	0.001		

Source: New York State, SPARCS, 2000–2002; reference categories in (parentheses).

DF, degrees of freedom; SE, standard error.

**Table 4.** Logistic Regression Predicting Revascularization Among Patients Age 45+ with Ischemic Heart Disease or Congestive Heart Failure, New York City, 2011–2013

	*B*	SE	Wald	DF	Sig	Exp. (*B*)	95% Confidence interval	
							Lower	Upper
Age	0.252	0.005	2663.394	1	0.000	1.286	1.274	1.299
Age squared	−0.002	0.000	3366.387	1	0.000	0.998	0.998	0.998
Female (male)	−0.431	0.010	1695.218	1	0.000	0.650	0.637	0.664
Non-Hispanic black (NH white)	−0.738	0.015	2328.647	1	0.000	0.478	0.464	0.492
Hispanic (NH white)	−0.241	0.015	245.419	1	0.000	0.786	0.763	0.810
Asian/other (NH white)	0.473	0.012	1475.788	1	0.000	1.605	1.567	1.644
Lowest income neighborhoods (highest income neighborhoods)	−0.215	0.015	205.496	1	0.000	0.806	0.783	0.831
Second quartile income neighborhoods (highest income neighborhoods)	−0.044	0.015	8.725	1	0.003	0.957	0.929	0.985
Third quartile income neighborhoods (highest income neighborhoods)	0.026	0.015	2.977	1	0.084	1.026	0.996	1.057
Uninsured (private insurance)	−0.679	0.033	422.249	1	0.000	0.507	0.475	0.541
Medicare (private insurance)	−0.546	0.014	1428.023	1	0.000	0.579	0.563	0.596
Medicaid (private insurance)	−0.529	0.015	1184.699	1	0.000	0.589	0.572	0.607
Other insurance (private insurance)	−1.530	0.110	194.128	1	0.000	0.216	0.175	0.268
Number of diagnoses on record	−0.084	0.001	5124.529	1	0.000	0.920	0.918	0.922
Total physicians 1000 population	0.000	0.000	0.629	1	0.428	1.000	1.000	1.000
Constant	−7.642	0.166	2131.061	1	0.000	0.000		

Source: New York State, SPARCS 2011–2013; reference categories in (parentheses).

We also examined race and insurance status. The odds of revascularization were about 75% lower among non-Hispanic blacks and 55% lower among Hispanic than non-Hispanic whites in 2000–2002 (Table 3). The odds were about 19% lower among Asians than among non-Hispanic whites in 2000–2002 (Table 3). During the 2011–2013 period, the odds of revascularization were about 52% lower among non-Hispanic blacks and 21% lower among Hispanic than non-Hispanic whites in 2000–2002. The odds were about 60% higher among Asians than among non-Hispanic whites (Table 4).

In 2000–2002, the odds of revascularization, for those without health insurance, were about 59% lower than among their insured counterparts. The odds were 37% lower among Medicaid recipients and 30% lower among Medicare beneficiaries, than among their privately insured counterparts (Table 3). Likewise, the odds were about 36% lower for those with “other” government health insurance. In 2011–2013, the odds of revascularization for persons without health insurance were about 49% lower than among their insured counterparts. In comparison with their privately insured counterparts, the odds were 41% lower among Medicaid recipients, 42% lower among Medicare beneficiaries, and about 78% lower for those with “other” government health insurance (Table 4).

## Discussion

As with disease prevalence, trends in the overall use of revascularization in NYC mirror national ones. Over the past 50 years, since the performance of the first CABG, treatment of obstructive CAD has changed. Indications for medical therapy versus surgical or percutaneous revascularization remain the subject of extensive discussion and debate. Guidelines from the American College of Cardiology Foundation/American Heart Association (ACCF/AHA) and the European Society of Cardiology/European Association for Cardiothoracic Surgery (ESC/EACTS) have provided standards of care for interventional cardiologists, cardiac surgeons, and the physicians who refer patients for these procedures. Important new issues covered in recent guidelines from the United States and Europe include the need for risk stratification before revascularization and the use of risk scores, with emphasis on the need to develop a multidisciplinary team-based approach.^18^ These guidelines also address the sensitive and controversial issues of self-referral and *ad hoc* PTCI, while providing detailed recommendations on the indications for revascularization versus optimal medical therapy for stable CAD, PTCI versus CABG for multivessel disease, and PTCI versus CABG for left main disease. Reasonable physicians caring for these patients may differ with respect to their assessments of available evidence and arguments for each management approach.^16^

Despite these important shifts in guidelines for revascularization and its role in the treatment of obstructive CAD, there continues to be good reason for concern about the overuse, misuse, and underuse of these treatments. There is evidence that decisions for proceeding with CABG or PTCI, in clinical practice, may not always follow international guidelines and appropriate use criteria.^19–21^ Although hospital administrative data are inadequate to reach conclusions about adherence to clinical guidelines, we have documented large and persistent disparities in the use of revascularization among patients hospitalized with IHD and CHD. These disparities call for further research.

Over the 2011–2013 period, non-Hispanic blacks and Hispanic whites were significantly less likely than non-Hispanic white patients to receive revascularization procedures in NYC. These racial and ethnic disparities were much smaller than those documented over the 2000–2002 period. It is not possible for us to determine whether the narrowing of these disparities is due to national^22^ or local interventions, but our results offer grounds for cautious optimism.

While our findings are encouraging, the persistence of gender, insurance status, and neighborhood-level disparities in the treatment of heart disease reinforces a looming policy concern on persistent inequalities in access to specialty services. With respect to gender disparities, many have argued that the prevailing conception of heart disease as a “man's disease” may explain gender differences in the treatment of heart disease.^23^ The implication of this position is to call for efforts to promote greater awareness among physicians and patients. In 2002, the National Heart, Lung, and Blood Institute (NHLBI), along with several national and community organizations, created *The Heart Truth* program to raise awareness of heart disease among women. Yet, even with these efforts, differences in the use of revascularization among men and women in NYC did not change by 2013. Because we rely on hospital administrative data, it is not possible to determine whether these differences can be explained by clinical factors, or respect for patients' values and preferences.

## Conclusions

Our findings point to the need for more clinical- and population-based research to improve understanding of why race/ethnicity, gender, insurance status, and neighborhood-level disparities persist in the treatment of heart disease even as the rates of heart disease have declined.

## Abbreviations Used

ACCF/AHA: American College of Cardiology Foundation/American Heart Association
AMI: acute myocardial infarction
CABG: coronary artery bypass graft
CAD: coronary artery disease
CHF: congestive heart failure
DF: degrees of freedom
DOHMH: Department of Health and Mental Hygiene
ESC/EACTS: European Society of Cardiology/European Association for Cardiothoracic Surgery
IHD: ischemic heart disease
NHLBI: National Heart, Lung, and Blood Institute
NYC: New York City
NYCCHS: New York City Community Health Survey
PTCI: percutaneous transluminal coronary intervention
SE: standard error
SPARCS: Statewide Planning and Research Cooperative System
TCNY: Take Care New York
